# Anemia is independently associated with mortality in people living with human immunodeficiency virus/acquired immune deficiency syndrome: A propensity score matching-based retrospective cohort study in China

**DOI:** 10.3389/fmed.2023.1055115

**Published:** 2023-02-15

**Authors:** Meihua Jin, Yanan Wang, Jing Li, Zhenqian Wu, Xiaoqi Liu, Hui Wang, Yuxin Chen, Ziyi Wang, Zhaowei Tong, Xiaofeng Li, Feilin Ren, Xiaojuan Zhu, Zhongrong Yang, Guangyun Mao

**Affiliations:** ^1^Huzhou Center for Disease Control and Prevention, Huzhou, Zhejiang, China; ^2^Division of Epidemiology and Health Statistics, Department of Preventive Medicine, School of Public Health & Management, Wenzhou Medical University, Wenzhou, Zhejiang, China; ^3^Center on Evidence-Based Medicine & Clinical Epidemiological Research, School of Public Health & Management, Wenzhou Medical University, Wenzhou, Zhejiang, China; ^4^Department of Infectious Diseases, Huzhou Central Hospital, Huzhou, Zhejiang, China; ^5^National Clinical Research Center for Ocular Diseases, Wenzhou, Zhejiang, China

**Keywords:** people living with HIV/AIDS, HIV/AIDS, mortality, anemia, propensity score matching

## Abstract

Although previous studies have suggested that hemoglobin is related to the health status of people living with human immunodeficiency virus/acquired immune deficiency syndrome (HIV/AIDS) (PLWHA), the role of anemia in mortality remains unclear. This study aimed to comprehensively quantify the effect of anemia on the mortality risk of PLWHA. In this retrospective cohort study, we thoroughly estimated the effect of anemia on PLWHA mortality, using data collected from January 2005 to June 2022 in the Huzhou area, in 450 subjects extracted from the database of the China Disease Prevention and Control Information System and matched them using a propensity score matching approach to balance potential confounding bias. The potential exposure–response relationship between anemia, hemoglobin concentration, and the mortality of PLWHA was also carefully estimated. A series of subgroup analyses, including interaction analysis, was further conducted to validate the robustness of the effect of anemia on PLWHA death risk. Anemia was significantly associated with an elevated death risk in PLWHA, with an increase of 74% (adjusted hazard ratio [AHR]: 1.74; 95% confidence interval [CI]: 1.03–2.93; *p* = 0.038) in those with anemia after adjusting for potential confounders. PLWHA with moderate or severe anemia had a higher risk of death, with an 86% increase (AHR = 1.86; 95% CI: 1.01–3.42; *p* = 0.045). Meanwhile, the AHR tended to increase by 85% on average (AHR = 1.85, 95% CI: 1.37–2.50; *p* < 0.001) with a per standard deviation (SD) decrease in plasma hemoglobin. Consistent relationships between plasma hemoglobin and the risk of death were further observed in the results from multiple quantile regression models, restricted cubic spline regression models, and a series of subgroup analyses. Anemia is an independent risk factor for HIV/AIDS-related mortality. Our findings may provide new insights into the relevance of PLWHA administration to public health policy, which demonstrate that this low-cost and routinely measured marker (hemoglobin) can be a marker of poor prognosis even before the start of HAART.

## 1. Introduction

Although a large number of financial resources and efforts have been put into the practice for its prevention and control over the past several decades, human immunodeficiency virus/acquired immune deficiency syndrome (HIV/AIDS) continues to be a major global public health issue (1–3). The number of people living with HIV/AIDS (PLWHA) is still huge, despite the wide application of the WHO 5Cs principles, which consist of consent, confidentiality, counseling, correct results, and connection with treatment and other services. According to the WHO, the prevalence of HIV/AIDS among adults aged 15–49 years was estimated as 0.7% (0.6–0.8%) at the end of 2021. Among the global population of 38.4 million (33.9–43.8 million) HIV cases, approximately 1.5 million (1.1–2.0 million) people were newly infected worldwide in 2021 (4).

People living with HIV/AIDS (PLWHA) generally transmit HIV to sexual partners, which increases the risk of co-infections (5). To control the HIV/AIDS epidemic more efficiently, a person who is diagnosed with HIV infection or is at an increased risk of acquiring HIV is recommended by the WHO to seek comprehensive and effective HIV prevention, testing, and therapy, especially antiretroviral treatment, as soon as possible along with periodic monitoring. Available evidence has demonstrated that highly active antiretroviral therapy (HAART) can efficiently reduce the incidence of opportunistic infections, decrease the mortality risk of PLWHA (6), and improve quality of life (7, 8). HAART can inhibit viral replication in the body, reduce the HIV viral load, and decrease the propensity of HIV transmission to others (9, 10). Nevertheless, HIV/AIDS may still play an important and non-negligible role in premature death in PLWHA. According to the WHO, 650,000 (510,000−860,000) people died of HIV-related illnesses worldwide in 2021. Previous studies have suggested that anemia is a major hematological complication of HIV infection and can lead to apparent fatigue, reduce PLWHA’s quality of life, and even endanger their life safety (11). Yesuf et al. reported that anemia can be detected in 30–80% of PLWHA cases and may increase the mortality of HIV/AIDS individuals (12).

Although a series of previous studies have reported that plasma hemoglobin is associated with the health status of PLWHA, whether anemia plays an independent role in HIV/AIDS-related death remains unclear (13–16). This knowledge is valuable for the efficient prevention and control of HIV/AIDS in practice. Therefore, we carefully designed and conducted this propensity score matching (PSM)-based retrospective cohort study to clarify this topic and emphasize the clinical and public health policy relevance of HIV/AIDS-induced premature death prevention and its clinical administration.

## 2. Materials and methods

### 2.1. Study participants

The data used in the present study were extracted from 1,694 PLWHA from the China Disease Prevention and Control Information System (CDPCIS) during 17 years of follow-up in the Huzhou area, China. Detailed information on participant enrollment, HAART, and follow-up can be found in a previous study (17). The inclusion criteria were as follows: over 18 years of age; living in the Huzhou area containing temporary residents; diagnosed or identified from 1 January 2005 to 30 June 2022; having completed baseline (pre HAART) laboratory serum testing; and being visited at least once. Survival time was determined as the duration from receiving HAART to death or 30 June 2022. PLWHA who received HAART before entering the cohort, those with missing baseline hemoglobin records, or those with illogical survival times were excluded from the final data analysis.

**Figure 1:**
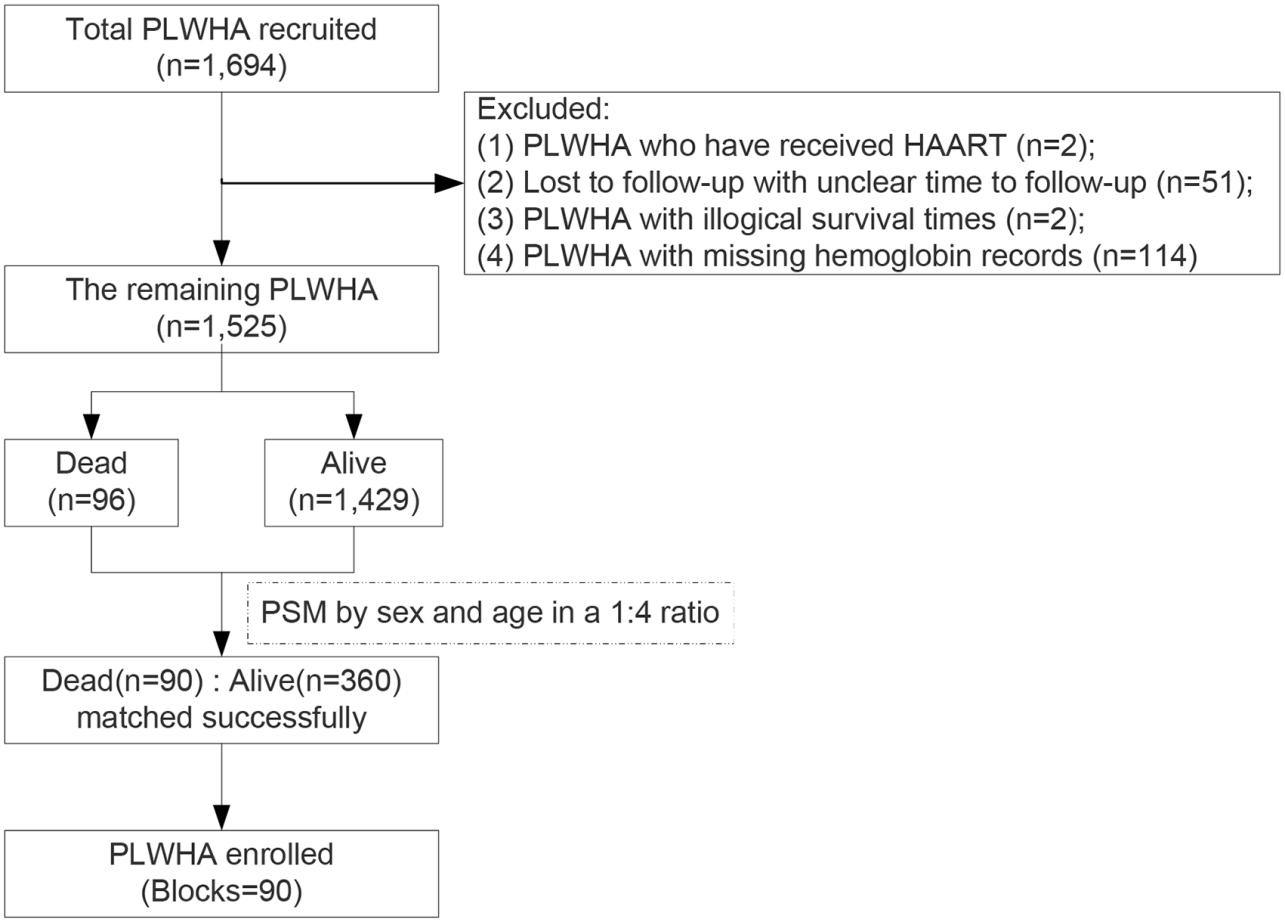
A flowchart of participants’ enrollment. PLWHA, people living with HIV/AIDS.

Among the 1,694 PLWHA enrolled in this study (Figure 1), 169 were excluded based on the inclusion criteria. Of the remaining 1,525 participants, 96 were dead and 1,429 were alive.

### 2.2. Study design

To efficiently control the potential impacts of known or unknown important confounders on the association of anemia with HIV/AIDS-related mortality in PLWHA, a PSM-based retrospective cohort study was conducted, in which the dead and alive PLWHA were matched by age and sex at a ratio of 1:4. A total of 450 participants (90 blocks containing 90 dead and 360 matched alive) were included in the final data analysis. The study protocol had been approved by the Ethics Committee of the Huzhou Center for Disease Control and Prevention (HZ2021001). All participants were notified of the purpose of this study before their enrollment and voluntarily participated. No information related to the subject’s privacy, including name, address, telephone, or other details, could be found in the final dataset.

### 2.3. Definition of anemia

According to the WHO diagnostic criteria, anemia is defined as a hemoglobin level of <130 g/L in males or < 120 g/L in females. To more comprehensively investigate the association of anemia with the death risk of PLWHA, anemia was further classified into two categories, in which hemoglobin from 110 to 129 g/L in males or 110 to 119 g/L in females was defined as mild anemia, and moderate or severe anemia was defined as hemoglobin <110 g/L (18).

### 2.4. Covariates

Data on the demographics and clinical characteristics of participants, including the following covariates, were extracted from the CDPCIS. In brief, information on age, sex, weight, height, marital status, education level, race, infection pathway, history of sexual behavior, whether coupled with sexually transmitted diseases (STDs), initial HAART prescription, disease stage (HIV or AIDS), WHO clinical stage, and other variables was collected strictly following the standard operating procedure of this study in a face-to-face interview or physical examination at enrollment. The body mass index (BMI) was calculated using the following equation: BMI = weight (kg)/height (m)/height (m). In addition, HIV/AIDS-associated laboratory testing, including CD4^+^ T lymphocytopenia (CD4), CD8^+^ T lymphocytopenia (CD8), serum hemoglobin, platelet (PLT), white blood cell (WBC), alanine aminotransferase (ALT), aspartate transaminase (AST), serum creatinine, total bilirubin (TBIL), and fasting plasma glucose (FPG), were carefully determined by experienced professional technicians from Huzhou CDC or local AIDS-designated treatment hospitals per standard clinical procedures and documented in the electronic medical records at CDPCIS.

### 2.5. Statistical analyses

Considering that high-quality data would be a solid foundation for credible conclusions, a series of necessary data cleanings and transformations, including outlier identification, logic error checking, and missing value imputation, were performed in advance to ensure satisfactory data quality in this study. First, outliers for continuous data were defined as beyond related mean ± 3*standard deviation (SD) for normal or similar normally distributed data or outside the scope of 0.5–99.5% when they were skewed. For categorical data, outliers were denoted as logic errors (i.e., the value of gender was neither male nor female). All outliers were verified and remeasured as much as possible to obtain their actual values or treated as missing values. Second, all variables with missing ratios greater than 30% were eliminated from the final working dataset. Otherwise, the missing values were filled using a 5-fold multiple imputation approach. Subsequently, sensitivity analysis on the comparison of pre-and post-imputation was additionally applied to validate the stability of imputations (Supplementary Table S1).

The effect of anemia on the death risk of PLWHA was assessed first using locally weighted regression models and restricted cubic spline regression models to observe the potential exposure–response relationship between mortality and serum hemoglobin concentration. It was then estimated in the following two ways: with exposure as a categorical variable (non-anemia vs. anemia) and as a continuous variable [scaled to standard deviation (SD) of hemoglobin reduction]. Furthermore, we examined the effect of decreased hemoglobin on HIV/AIDS-related mortality in PLWHA based on multiple quantile regression models (quartiles). Multivariable Cox proportional hazard regression models were used to estimate the independent association of anemia or serum hemoglobin level with the mortality of PLWHA, in which potential impacts due to covariates (*p*-value < 0.2) in the univariate analysis were adjusted. In addition, to verify the consistency of the effect of anemia on the death risk of PLWHA, a series of subgroup analyses containing interaction effects were performed.

All data management and statistical analyses were performed using R Version 4.2.0 (Copyright^©^ 2022 The R Foundation for Statistical Computing) with a package of mice 3.14.0, survminer 0.4.9 and survival 3.3–1, rms_6.3–0, ggpubr 0.4.0, and ggplot2 3.3.6. Significance was set at a *p*-value ≤ 0.05.

## 3. Results

### 3.1. Participants characteristics

The mortality rate of the 1,694 PLWHA was 6.08% (81.6% for men and 18.4% for women). In this PSM-based retrospective cohort study, the mean age of 450 PLWHA included in this study was 48.5 years (median: 47.0 years). Among them, 82.2% (370/450) were men, 89 had anemia at baseline (pre HAART), and 90 died during follow-up. When comparing characteristics by status (Table 1), dead PLWHA were more likely to have a lower BMI; a higher proportion of unmarried, divorced, or widowed individuals; and a smaller percentage of patients receiving combined HAART, including tenofovir disoproxil fumarate (TDF) + efavirenz (EFV) + lamivudine (3TC). Meanwhile, they also tended to be categorized to have AIDS or be over the WHO III clinical stage, had heterosexual transmission experience, liver dysfunction, and were less likely to have normal CD4^+^ T-lymphocyte count, hemoglobin, platelets, and white blood cell levels when compared with the live PLWHA.

**Table 1:** Demographics and clinical characteristics of PLWHA.

Variables	Alive (*n* = 360)	Dead (*n* = 90)	*P*
Age, years	47.0 (40.0, 59.0)	47.0 (40.0, 59.0)	0.991
Gender			0.810
Male	292 (81.1)	72 (80.0)	
Female	68 (18.9)	18 (20.0)	
BMI, kg/m^2^	22.1 (20.6, 23.9)	21.0 (19.5, 23.2)	0.009
Marital status			0.015
Married	242 (67.2)	46 (51.1)	
Unmarried	45 (12.5)	15 (16.7)	
Divorced or widowed	73 (20.3)	29 (32.2)	
Education category			0.422
Illiterate or elementary school	147 (40.8)	39 (43.3)	
Junior or high or secondary school	177 (49.2)	46 (51.1)	
College and above	36 (10.0)	5 (5.6)	
Race			0.143
Han	350 (97.2)	84 (93.3)	
Others	10 (2.8)	6 (6.7)	
Infection pathway			0.067
Heterosexual transmission	238 (66.1)	69 (76.7)	
MSM	94 (26.1)	13 (14.4)	
Others	28 (7.8)	8 (8.9)	
Whether coupled with STDs			0.002
No	269 (74.7)	58 (64.4)	
Yes	69 (19.2)	16 (17.8)	
Unknown	22 (6.1)	16 (17.8)	
Initial HAART prescription			<0.001
TDF + EFV + 3TC	204 (56.7)	26 (28.9)	
3TC + AZT + EFV	67 (18.6)	16 (17.8)	
NVP + 3TC + AZT	39 (10.8)	26 (28.9)	
Others	50 (13.9)	22 (24.4)	
Disease stage			<0.001
HIV	195 (54.2)	24 (26.7)	
AIDS	165 (45.8)	66 (73.3)	
WHO clinical stage			<0.001
I or II	352 (97.8)	80 (88.9)	
III or IV	8 (2.2)	10 (11.1)	
CD4, cells/L	222.7 (143.0, 315.0)	136.5 (40.6, 252.0)	<0.001
CD8, cells/L	681.6 (530.6, 923.0)	635.6 (484.3,864.9)	0.145
Hemoglobin, g/L	139.0 (125.0, 151.0)	131.5 (110.0, 140.0)	<0.001
Platelet, 10^9^/L	180.0 (136.5, 218.5)	161.0 (122.0, 203.0)	0.008
WBC, 10^9^/L	4.9 (4.0, 6.2)	4.5 (3.6,5.7)	0.012
ALT, U/L	20.7 (15.0, 31.0)	24.6 (16.0, 32.0)	0.201
AST, U/L	22.6 (19.0, 29.8)	26.8 (22.0, 35.0)	<0.001
Creatinine, μmol/L	72.5 (63.7, 82.0)	73.0 (63.0, 84.4)	0.761
TBIL, μmol/L	10.1 (7.8, 13.3)	9.4 (7.3, 12.5)	0.130
FPG, mmol/L	5.4 (4.9, 5.9)	5.4 (4.9, 6.0)	0.997

Continuous variables were described as median (first and third quartiles) as its distribution was skewed and the Mann–Whitney *U*-test was applied to compare the difference between the two groups; Categorical data were presented with a number (%), and chi-square tests or Fisher’s exact test was used to compare the differences between alive and dead participants. MSM, men who have sex with men; STDs, sexually transmitted infections; BMI, Body mass index; CD4, CD4^+^ T-lymphocyte count; CD8, CD8^+^ T-lymphocyte count; WBC, White blood cell; ALT, Alanine aminotransferase; AST, Aspartate transaminase; TBIL, Total bilirubin; FPG, Fasting plasma glucose; TDF, tenofovir disoproxil fumarate; EFV, efavirenz; 3TC, lamivudine; AZT, Zidovudine; NVP, Nevirapine.

### 3.2. Independent association of anemia and hemoglobin with mortality

**Figure 2:**
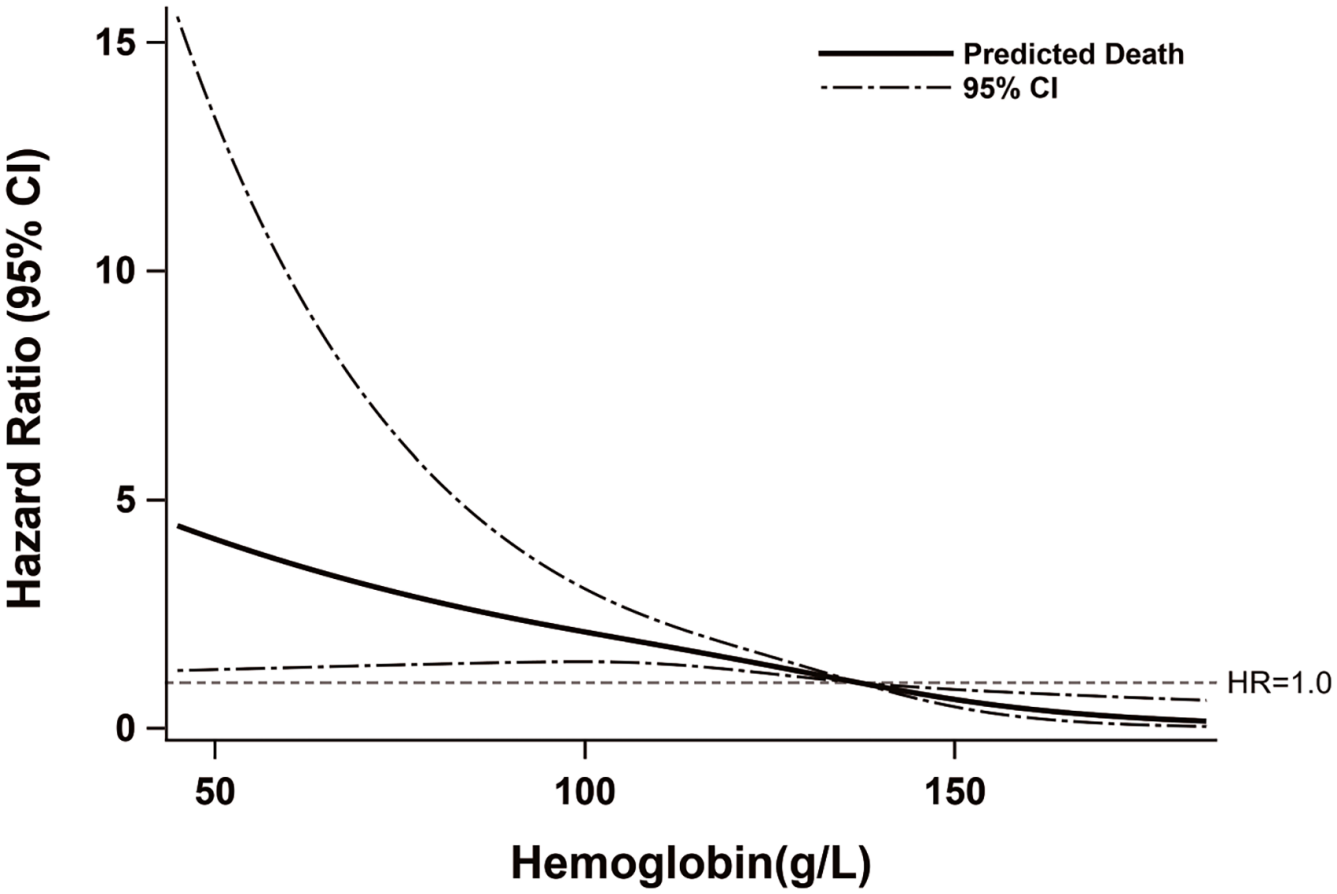
Exposure–response relationship between hemoglobin and the mortality of PLWHA.

According to the results of a locally weighted regression model (Figure 2), the mortality risk of PLWHA was associated with the hemoglobin level. This revealed that an obvious negative exposure–response relationship between participants’ serum hemoglobin levels and their death risk might exist. A very similar relationship between exposure and outcome was additionally observed depending on a restricted cubic spline regression model-based result (Supplementary Figures S1, S2). The proportion of deaths in participants with anemia (30.53%) was higher than that in PLWHA without anemia (15.67%). After adjusting for the covariates (BMI, CD4, AST, TBIL, WBC, Race, whether infected with STDs, initial HAART prescription, marital status, disease stage, WHO clinical stage, infection pathway, and platelet) with *p*-value of <0.2 (Table 1), the death risk for PLWHA suffering from anemia was significantly increased by 74% (adjusted hazard ratio [AHR]:1.74; 95% confidence interval [CI]:1.03–2.93; *p* = 0.038) when compared with those without anemia (Table 2). Meanwhile, as compared to PLWHA without anemia, AHR (95% CI) of death for those with mild and moderate or severe anemia were 1.60 (0.84–3.07, *p* = 0.153) and 1.86 (1.01–3.42, *p* = 0.045), respectively. Furthermore, the association of mortality in PLWHA with hemoglobin level was quantified. With a per standard deviation decrease in hemoglobin level, the death risk increased by 85% on average (AHR: 1.85; 95%CI: 1.37–2.50; *p* < 0.001). When compared to PLWHA with the highest quartile of hemoglobin (≥150 g/L), the death risk for those in the third (138–149 g/L), second (123 to 137 g/L), and first (<123 g/L) quartiles of serum hemoglobin was significantly increased by 216% (AHR:3.16;95% CI:1.34–7.42; *p* = 0.008), 217% (AHR:3.17; 95% CI:1.32–7.62; *p* = 0.010), and 298% (AHR:3.98; 95% CI:1.62–9.79, *p* = 0.003), respectively. A significant negative linear relationship between serum hemoglobin and mortality in PLWHA was detected (*p*-value for trend = 0.006). These results strongly indicate that anemia is an independent risk factor for death in PLWHA.

**Table 2:** Effect of anemia and hemoglobin on the mortality of PLWHA.

Exposure	*n*	Death, ^#^ (%)	Crude		Adjusted^#^	
			*HR(95%CI)*	*P*	*HR(95%CI)*	*P*
Anemia						
Strata 1						
No	319	50 (15.67)	Ref.		Ref.	
Yes^*^	131	40 (30.53)	2.21 (1.45, 3.35)	<0.001	1.74 (1.03, 2.93)	0.038
Strata 2						
Normal	319	50 (15.67)	Ref.		Ref.	
Mild^&^	73	19 (26.03)	1.91 (1.13, 3.24)	0.017	1.60 (0.84, 3.07)	0.153
Moderate or severe^$^	58	21 (36.21)	2.58 (1.54, 4.31)	<0.001	1.86 (1.01, 3.42)	0.045
Hemoglobin, g/L						
Per SD reduction	450	90 (20.0)	1.94 (1.53, 2.46)	<0.001	1.85 (1.37, 2.50)	<0.001
Quartiles						
Q_4_ (≥150)	112	8 (7.14)	Ref.			Ref.
Q_3_ (138–149)	111	23 (20.72)	3.23 (1.44, 7.22)	0.004	3.16 (1.34, 7.42)	0.008
Q_2_ (123–137)	114	23 (20.18)	3.38 (1.51, 7.56)	0.003	3.17 (1.32, 7.62)	0.010
Q_1_ (<123)	113	36 (31.86)	5.34 (2.48, 11.51)	<0.001	3.98 (1.62, 9.79)	0.003
*P* for trend				<0.001		0.006

^*^Anemia is defined as hemoglobin levels of less than 130 g/L in males or hemoglobin levels of less than 120 g/L in females. ^&^Mild anemia is defined as hemoglobin levels of 110–129 g/L in males or hemoglobin levels of 110–119 g/L in females. ^$^Moderate or severe anemia is defined as hemoglobin levels of less than 110 g/L. ^#^Adjusted for BMI, CD4, AST, TBIL, WBC, race, whether infected with STDs, initial HAART prescription, marital status, disease stage, WHO clinical stage, infection pathway, and platelet.

### 3.3. Consistency of the effect of anemia on mortality

To further assess the consistency of the independent effect of serum hemoglobin on the risk of death in PLWHA, a series of subgroup analyses, including interaction analysis, were performed. As shown in Table 3, the effect of anemia on the death risk of PLWHA was consistent and evident, especially in males and participants with a higher BMI, CD4^+^ T-lymphocyte count (CD4), lower white blood cell count (WBC), total bilirubin (TBIL), normal alanine aminotransferase (ALT), and fasting plasma glucose (FPG). These results indicated that the independent effect of anemia on PLWHA death would be more evident in males and overweight or obese participants. It also tended to be masked by impaired immune function, liver function damage, and diabetes to some extent, although there was no obvious interaction between anemia and the aforementioned covariates.

**Table 3:** Subgroup analyses on the association of anemia with the mortality of PLWHA.

Variables	*N*	Death, *n*(%)	*HR* (95%CI)	*P* for interaction
Gender				0.622
Male	364	72 (19.8)	2.46 (1.30, 4.68)	
Female	86	18 (20.9)	1.10 (0.21, 5.86)	
Age, years				0.591
<47	220	44 (20.0)	1.59 (0.65, 3.87)	
≥47	230	46 (20.0)	1.52 (0.70, 3.34)	
BMI, kg/m^2^				0.623
<24	351	72 (20.5)	1.95 (1.09, 3.50)	
≥24	99	18 (18.1)	0.92 (0.17, 4.91)	
CD4, cell/μL				0.367
<200	215	58 (27.0)	1.23 (0.64, 2.35)	
≥200	235	32 (13.6)	3.63 (1.08, 12.2)	
WBC, 109/L				0.960
<4.9	224	54 (24.1)	1.80 (0.89, 3.64)	
≥4.9	226	36 (15.9)	1.39 (0.47, 4.10)	
Creatinine, μmol/L				0.931
<72.65	225	44 (19.6)	1.81 (0.75, 4.33)	
≥72.65	225	46 (20.4)	1.21 (0.51, 2.89)	
TBIL, umol/L				0.172
<9.89	225	50 (22.2)	1.49 (0.71, 3.15)	
≥9.89	225	40 (17.8)	1.76 (0.72, 4.34)	
ALT, U/L				0.411
<40.0	222	41 (18.5)	2.09 (1.12, 3.90)	
≥40.0	228	49 (21.5)	10.37 (0.02, 6287.04)	
FPG, mmol/L				0.072
<6.1	224	46 (20.5)	2.27 (1.25, 4.13)	
≥6.1	226	44 (19.5)	1.19 (0.16, 9.20)	

BMI, Body mass index; CD4, CD4 T-lymphocyte count; WBC, White blood cell; TBIL, Total bilirubin; ALT, Alanine aminotransferase; FPG, Fasting plasma glucose.

## 4. Discussion

Our findings demonstrate that moderate or severe anemia is a non-negligible independent risk factor for HIV/AIDS-related death in PLWHA, especially in male PLWHA patients and those with lower BMI, impaired immune function, liver function damage, and normal fasting plasma glucose. The propensities of AIDS and some opportunistic diseases in dead PLWHA were also higher than those of alive ones.

Previous studies have reported that HIV infection-related health impairments, including opportunistic infections, tumors, and malnutrition will not only reduce the quality of life of PLWHA but are also be affected by factors such as sex, age, economic status, marital status, educational level, CD4^+^ T cell count, and viral load (19–21). Available evidence reveals that anemia is quite common in PLWHA and is significantly associated with an increased risk of premature death (22). Therefore, the actual effect of anemia on the risk of death from PLWHA should be comprehensively assessed. Consistent with our results, two prior studies have reported that lower baseline plasma hemoglobin levels may be an independent marker of the risk of death in PLWHA (13, 14). Another study indicated that anemia might be a predictor of AIDS-related mortality (23). Shen et al. also suggested that anemia is associated with HIV infection in newly reported patients with HIV/AIDS, and its severity is also significantly related to the reduction in their CD4 counts (24). Based on this PSM-based retrospective cohort study, our results demonstrate that anemia or a low concentration of serum hemoglobin is an independent risk factor for mortality in PLWHA.

It is well known that HIV may attack an individual’s immune system by destroying CD4 cells and weakens immunity against important opportunistic infections, including tuberculosis, severe bacterial infections, and some cancers. As a major component of red blood cells (RBC), hemoglobin relies on ferritin in the center of RBC to undertake oxygen transport and plays a critical role in maintaining the normal function of RBC (25). It has also been reported that the RBCs of the participants will also be affected by HIV infection. This may be one of the potential mechanisms by which anemia or an obvious decrease in serum hemoglobin is prevalent in PLWHA. Another study suggested that anemia may have resulted from insufficient production of erythropoiesis-stimulant erythropoietin in the kidney (26). Subsequently, it is noteworthy that ferritin is an important iron-containing protein (27). Although the lack of ferritin also induces anemia or decreased hemoglobin, it does not change the relationship between anemia and HIV/AIDS-induced health damage. Therefore, timely identification of anemia and assessment of its severity in PLWHA would be very valuable for the effective prevention and control of HIV/AIDS-related health impairments, including premature death. Therefore, beyond routine HAART, appropriate iron supplements (such as ferrous glucose and ferrous sulfate) or dietary intervention (such as animal offal, fish, cherries, and tomatoes) should also be provided to PLWHA, especially those with apparent anemia, as early as possible. These measures will be beneficial to greatly increase hemoglobin concentration, significantly improve anemia, and reduce the probability of mortality risk in PLWHA to a large extent. This assumption should be further validated by additional experimental studies, especially large-scale, multi-center, randomized, double-blind clinical trials (RCT).

Our findings were dependent on a population-based retrospective cohort study rather than a case–control or descriptive study. A cohort study provides better evidence to reveal the actual causal relationship between a specific exposure and outcome and avoid a potentially confused cause-and-effect relationship when compared with other observational studies. The independent effect of hemoglobin on the death risk of PLWHA has been thoroughly and comprehensively analyzed based on locally weighted regression models, restricted cubic spline regression models, multivariable logistic regression models, and a series of subgroup assessments, including interaction analysis.

The present study also had some limitations. First, the current study included only 90 dead and 360 live PLWHA. This will inevitably lead to concerns regarding the potentially insufficient sample size. However, the small sample size does not necessarily mean that it is insufficient. In general, the insufficient sample size will result in inadequate power, in which significant findings will not be detected to a great extent. Fortunately, the results of this study revealed a significant effect of anemia on the death risk of PLWHA. Although the current sample size is not very large, it does not affect the reliability of our findings. Second, all variables used in the present study were derived from baseline (pre HAART) data, and some of their values may have changed during the follow-up. This may partly induce additional bias and potentially affect the strength of the effect of anemia. In addition, a probable source of selection bias may be induced by “only completed baseline laboratory” and “missing baseline hemoglobin records” since individuals with symptoms are more likely to obtain these data (i.e., laboratory indicators such as hemoglobin). Therefore, this really may cause selection bias to some extent. In the present study, only 6.7% (114/1694) PLWHA missed baseline hemoglobin and those with and without hemoglobin are highly comparable in the further comparison, which indicates that the hemoglobin is missed completely at random (MCAR) and may not induce obvious selection bias on our findings.

## 5. Conclusion

The present study demonstrates that anemia is an independent risk factor for mortality in PLWHA receiving HAART, which strongly indicates that this low-cost and routinely measured marker (hemoglobin) may be a potential indicator of PLWHA’s prognosis. The effect of anemia on the death risk of PLWHA is affected by sex, obesity, immune function, liver function, and fasting plasma glucose levels. Our findings may provide new insights into the relevance of PLWHA administration to public health policy.

## Data availability statement

The raw data supporting the conclusions of this article will be made available by the authors, without undue reservation.

## Ethics statement

The studies involving human participants were reviewed and approved by the Ethics Committee of Huzhou Center for Disease Control and Prevention. Written informed consent for participation was not required for this study in accordance with the national legislation and the institutional requirements.

## Author contributions

MJ, JL, XQL, and ZY: conceptualization, funding acquisition, supervision, draft, and editing. YW, HW, YC, and ZYW: conceptualization, data management, data analysis, and methodology. ZT, XFL, ZQW, FR, and XZ: conducting a research and investigation process, data management, and data collection. GM: conceptualization, data management, data analysis, methodology, writing, reviewing, editing, and supervision. All authors reviewed and approved the final manuscript.

## Funding

This study was supported by the Medical and Health Research Project of Zhejiang Province (2022KY369), the Huzhou Medical Key Supporting Discipline (Epidemiology), and the Key Laboratory of Emergency detection for Public Health of Huzhou.

## Conflict of interest

The authors declare that the research was conducted in the absence of any commercial or financial relationships that could be construed as a potential conflict of interest.

## Publisher’s note

All claims expressed in this article are solely those of the authors and do not necessarily represent those of their affiliated organizations, or those of the publisher, the editors and the reviewers. Any product that may be evaluated in this article, or claim that may be made by its manufacturer, is not guaranteed or endorsed by the publisher.
