# Supplementary material for: Anemia is independently associated with mortality in people living with human immunodeficiency virus/acquired immune deficiency syndrome: A propensity score matching-based retrospective cohort study in China
Source: Front Med (Lausanne). 2023 Feb 15;10:1055115. doi: 10.3389/fmed.2023.1055115 (PMC9975156; doi:10.3389/fmed.2023.1055115)
Supplement: Supplementary file 1 [file Data_Sheet_1.pdf]

Supplementary Material

1 Supplementary Tables

Supplemental Table S1. Comparison of pre- and post-imputations by 5-fold multiple imputation

Variables	Pre-imputations	Post-imputations	*P value*
**STDs**			1.000
None	1131(74.2)	1132(74.2)	
Yeath	274(18.0)	274(18.0)	
Unknown	119(7.8)	119(7.8)	
**WHO clinical stage**			0.998
I or II	1486(97.5)	1487(97.5)	
III or IV	38(2.5)	38(2.5)	
**ALT, U/L**	22.0(15.0,33.0)	22.0(15.1,33.0)	0.765
**AST, U/L**	22.0(18.4,29.0)	22.4(18.8,29.6)	0.197
**CD4, cells/L**	244.2(142.0,347.0)	232.0(137.6,335.0)	0.158
**Platelet, 10^9/L**	183.0(144.0,221.0)	183.0(144.0,220.0)	0.917
**WBC, 10^9/L**	5.1(4.2,6.2)	5.1(4.2,6.2)	0.728
**Creatinine, mmol/L**	72.6(64.0,84.0)	72.6(64.5,83.0)	0.885

**TBIL, mmol/L**	9.9(7.3,13.4)	10.0(7.7,13.0)	0.421
**FPG, mmol/L**	5.3(4.8,5.8)	5.3(4.9,5.8)	0.347

Note: *Continuous variables was described as median (1st quartile, 3rd quartile) as its distribution was skewed and Mann-Whitney U test was applied to compare the difference between two groups; Categorical data were presented with number (%) and chi-square tests or Fisher's exact test were used to compare the differences between pre- and post-imputations data.*

STDs: sexually transmitted infections; CD4: $CD4^+$ T-lymphocyte count; WBC: White blood cell; ALT: Alanine aminotransferase; AST: Aspartate transaminase; TBIL: Total bilirubin; FPG: Fasting plasma glucose.

2 Supplementary Figures

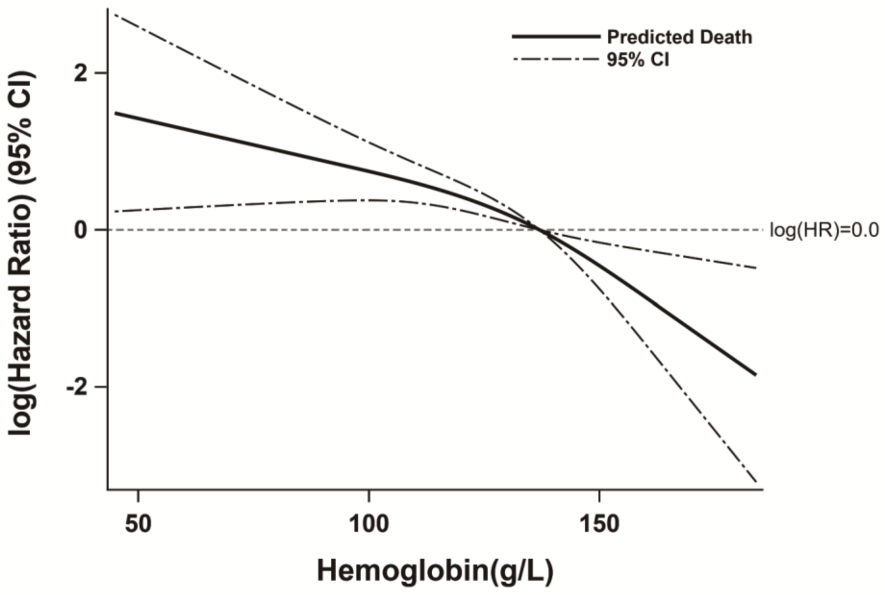

Supplemental Figure S1. Predictive effect of hemoglobin on the mortality risk of participants

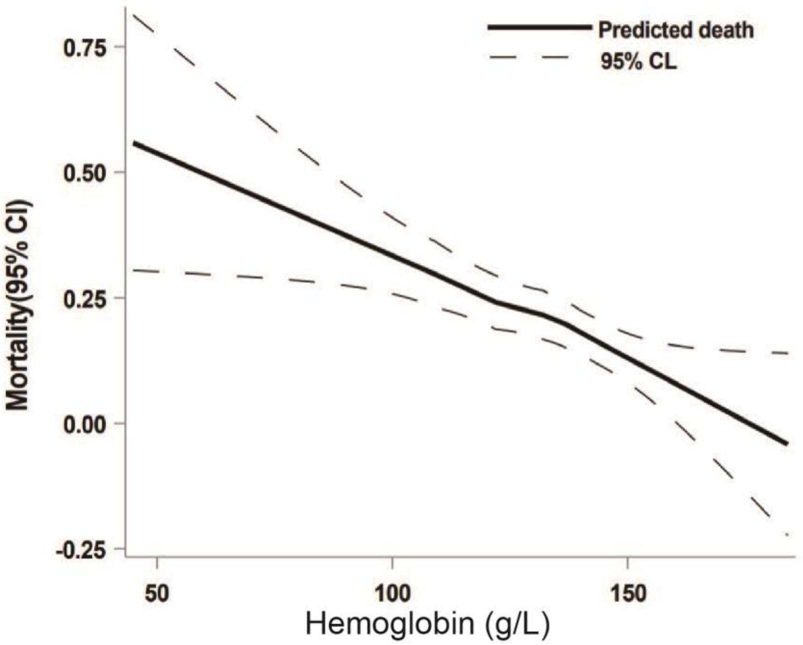

Supplemental Figure S2. Predictive effect of hemoglobin on the mortality risk of participants
